# Risk analysis of maize yield losses in mainland China at the county level

**DOI:** 10.1038/s41598-020-67763-3

**Published:** 2020-06-30

**Authors:** Xuan Li, Shibo Fang, Dong Wu, Yongchao Zhu, Yingjie Wu

**Affiliations:** 1 State Key Laboratory of Sever Weather, Chinese Academy of Meteorological Sciences, Beijing, 100081 China; 2 Collaborative Innovation Centre on Forecast and Evaluation of Meteorological Disasters, Nanjing University of Information Science & Technology, Nanjing, 210044 China; 3 Academy of Applied Meteorology, Nanjing University of Information Science & Technology, Nanjing, 210044 China; 4 Meteorological Observation Center, China Meteorological Administration, Beijing, 100081 China

**Keywords:** Climate sciences, Ecology, Natural hazards

## Abstract

Food security in China is under additional stress due to climate change. The risk analysis of maize yield losses is crucial for sustainable agricultural production and climate change impact assessment. It is difficult to quantify this risk because of the constraints on the high-resolution data available. Moreover, the current results lack spatial comparability due to the area effect. These challenges were addressed by using long-term county-level maize yield and planting area data from 1981 to 2010. We analyzed the spatial distribution of maize yield loss risks in mainland China. A new comprehensive yield loss risk index was established by combining the reduction rate, coefficient of variation, and probability of yield reduction after removing the area effect. A total of 823 counties were divided into areas of lowest, low, moderate, high, and highest risk. High risk in maize production occurred in Heilongjiang and Jilin Provinces, the eastern part of Inner Mongolia, the eastern part of Gansu-Xinjiang, west of the Loess Plateau, and the western part of the Xinjiang Uygur Autonomous Region. Most counties in Northeast China were at high risk, while the Loess Plateau, middle and lower reaches of the Yangtze River and Gansu-Xinjiang were at low risk.

## Introduction

Crop production has increased rapidly over the past several decades but with significant variations across the world^1,2^. Increases in crop production are mainly due to technological developments, infrastructure improvements, and investment increases, such as increases in fertilizer investment, especially after 2003 in China, while climate conditions predominantly induce instability in crop production, which could explain 30% or more of the variations in the global crop yield^3–5^.

China suffers from climate change and severe agro-meteorological disasters, including drought and floods^6^. The precipitation distribution exhibits regional differences; the amount of precipitation varies dramatically from less than 100 mm/year to more than 1,000 mm/year^7^. It has been estimated that the proportion of the area in China affected by drought disasters will increase with global warming, from 15.4 to 44.00% by 2100^3^. Crop production in China significantly depends on irrigation infrastructure, especially in poverty areas^8^. All these problems and challenges place additional stress on food security throughout China^2^.

The assessment of the risks of crop yield losses caused by climatic and socioeconomic conditions is crucial for sustainable agricultural production and inquiries regarding the uncertainty and risk associated with climate change^9,10^. To date, extensive research has been conducted on crop production risks, mainly focusing on the content, methods, indicators, and technologies used for assessment^3,11,12^. Due to the complexity of crop systems and yield formation^13,14^, when analyzing the impacts of climatic and socioeconomic conditions on crop production, most studies have focused on one or a combination of several meteorological factors (temperature, sunshine hours, and precipitation)^12,15,16^ or used crop-climate models to describe the responses of crops^17–20^. However, the observed crop yield is the result of the interaction of nature, crop genotype, and socioeconomic components, such as the selected crop varieties and planned management level^21^. The accuracy of an assessment could decrease due to the uncertainties in the physiological, ecological and parameterization processes^21^ described in the model, and the failure of the model to include all of the interactions because of the complexity in yield-determining processes^22,23^. Only some practices and crop systems can be simulated by models with confidence, rather than all of the crop production under the various socioeconomic or climatic environments^23^. The yield loss risk, which is estimated by the method proposed in IPCC 5th assessment reports, is determined by three indicators: exposure degree, sensitivity, and adaptive capacity^24^. Many researchers have selected a method that directly quantifies the risk of crop yield loss based on historical time series yield data^25–27^, which could integrate climatic conditions and socioeconomic components, as these factors are directly reflected in the data. Various indices, which include the coefficient of variation (CV) and yield reduction rate, have been commonly used to indicate the risk of losses^25,27^. However, assessments are often conducted at a coarse resolution, such as at the provincial or district level^6^, or at a high resolution over small regions^27,28^, which has significant limitations because of the difficulty in accessing yield data at high spatial resolution. In addition, these studies focused on only the economic responses to sown area size or used the percentage of the affected areas to planting areas to show the degree of damage/exposure^24,28–30^, without considering the area effects (see the next paragraph for further explanation). These choices induced the lack of spatial comparability in the results.

**Figure 1:**
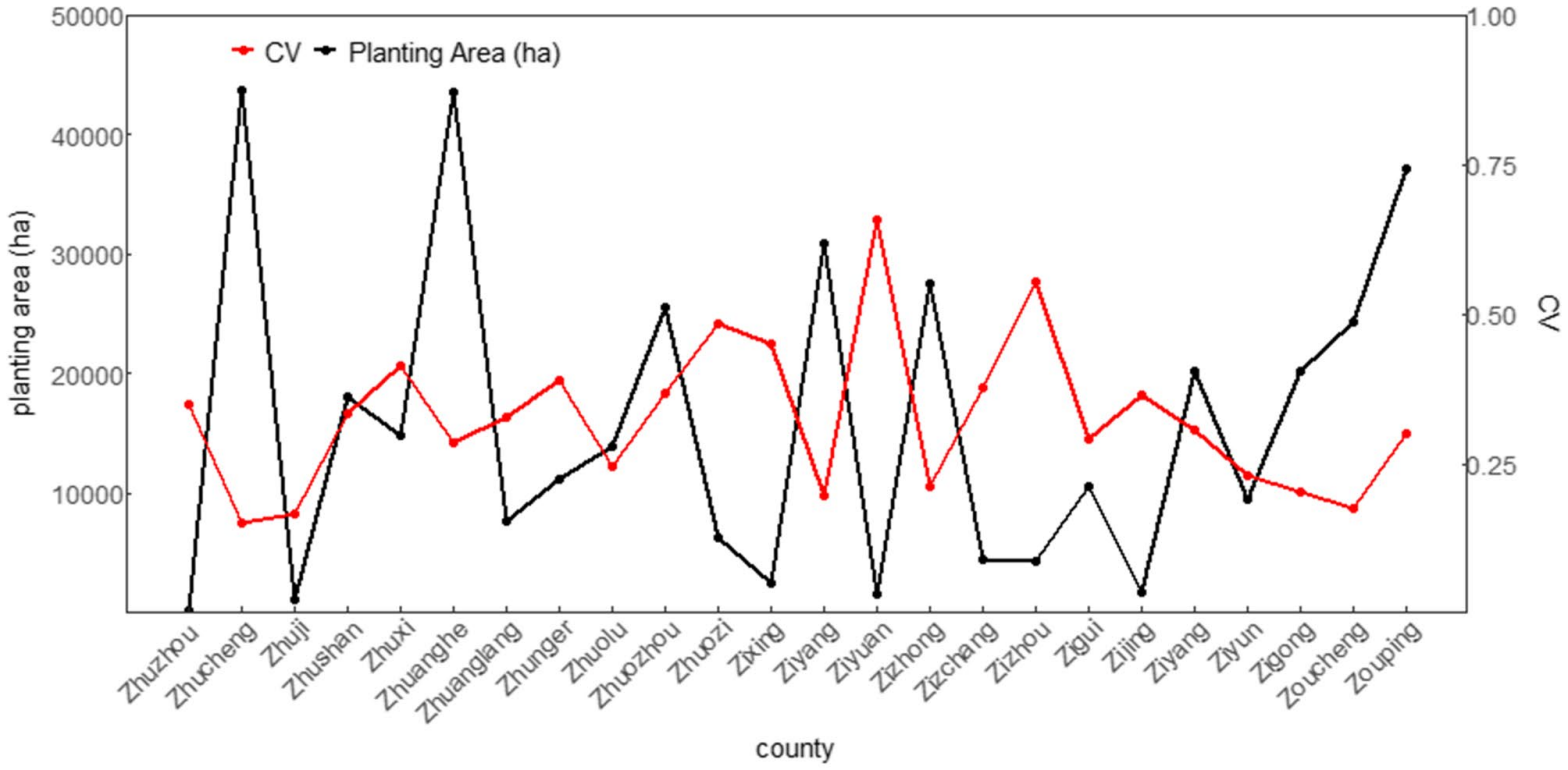
Coefficient of variation ($\mathrm{C}\mathrm{V}$) of the annual yield per unit area of maize for 24 counties with different planting areas from 1981 to 2010. The $\mathrm{C}\mathrm{V}$ (red line with dots) is calculated by using Eq. (1). The planting area (black line with dots) is calculated by the temporal average. The Pearson's pairwise correlation coefficient is − 0.53. MATLAB software^32^ version R2016a (https://cn.mathworks.com/products/matlab/) was used to draw the plot.

Most of the farmland in China is distributed in the eastern region, including Heilongjiang, Hebei, Henan, and Shandong Provinces, which host large crop-planting areas in most counties^31^. The planting scale of a county affects the yield loss risk result when field-based or farm-based observed yields are aggregated by county. For instance, the increase/decrease of the yield in some fields will offset the decrease/increase in other fields when the field yields are summed, and then the fluctuation or variation in yield will be underestimated. Moreover, the increasing crop area is threatening food security from increased competition for land for food production^23^. Thus, the actual risk should be higher. In contrast, the provinces in western China, such as Qinghai Province, the Tibet Autonomous Region, and the Xinjiang Uygur Autonomous Region (Xinjiang), have relatively small crop-planting areas in most counties^31^, where the estimated yield variation or loss risk could be higher than those of the counties with large planting areas. This suggests that the risk of small crop-planting areas could be overestimated in this area. Twenty-four counties (in western and eastern China) were randomly selected from the alphabetically ordered data of maize yield and planting area as a case study to test the hypothesis (Fig. 1). The CV of the annual yield per unit area of maize from 1981 to 2010 was calculated for each of the 24 counties to reflect the variation in maize yield. The Pearson’s pairwise correlation coefficient between planting area and CV was − 0.53, which indicates that there is a strong negative relationship between planting area and CV. The influence of planting area scale, which induced the overestimation or underestimation of yield loss risk, is referred to as the “area effect”. This study is the first to consider the area effect in the analysis of yield loss risk. The area effect will be removed or mitigated by introducing the indicator of the standardized planting area in our study.

This study aims to provide high-resolution information on the spatial distribution of yield loss risk based on a new comprehensive risk index, which was established by combining the reduction rate, CV, and probability of yield reduction after removing the area effect. The proposed index can improve the spatial comparability of risk. The results may be crucial for agricultural decision-support systems and climate change assessments.

## Result

### Distribution of the coefficient of variation ($\mathbf{C}\mathbf{V}$)

**Figure 2:**
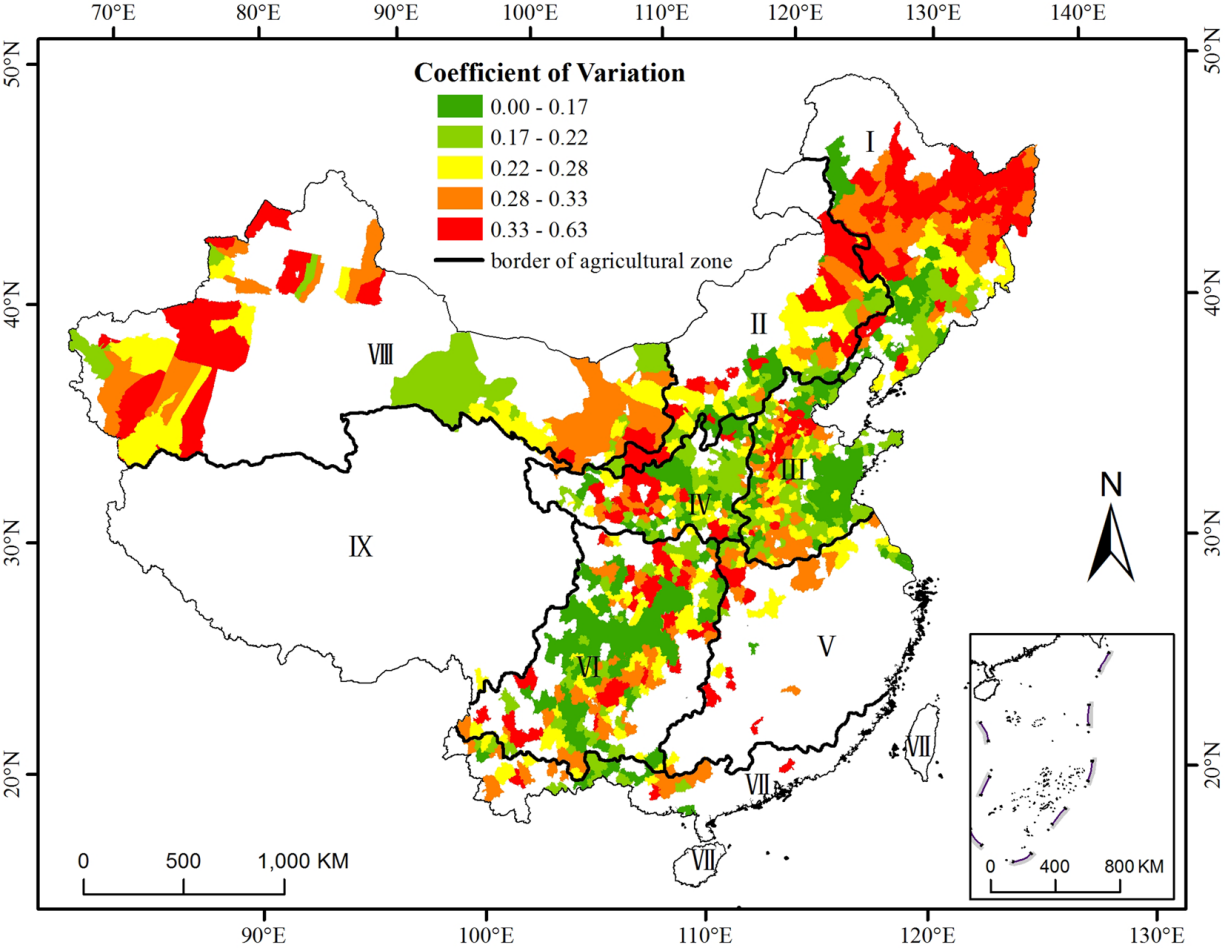
Distribution of the $\mathrm{C}\mathrm{V}$ for maize in the main production areas in China from 1981 to 2010. The data were processed in MATLAB software^32^ version R2016a (https://cn.mathworks.com/products/matlab/). The map was generated with ESRI ArcGIS software^33^ version 10.2.1 (URL: https://www.esri.com/software/arcgis/arcgis-for-desktop).

The $\mathrm{C}\mathrm{V}$ indicates the stability of maize production. A high $\mathrm{C}\mathrm{V}$ means that the maize yield fluctuates greatly between years and that the yield is vulnerable to both climatic and socioeconomic conditions. The $\mathrm{C}\mathrm{V}$ identified three regions with high variations covering (1) parts of Heilongjiang and Jilin Provinces and the eastern part of the Inner Mongolia Autonomous Region (Inner Mongolia); (2) the eastern part of zone VIII and western part of zone VI, including parts of Shanxi Province and the Ningxia Hui Autonomous Region; and (3) the western part of agricultural zone VIII (Fig. 2). The largest fluctuations occurred in the northern part of zone I, covering part of Heilongjiang Province, and small fluctuations occurred in the southern part of zone I, covering part of Liaoning Province. In contrast, the eastern part of the zone III covering parts of Shandong Province, Jiangsu Province and Anhui Province, and the southwestern region in China, covering Sichuan Province and Chongqing city, have low $\mathrm{C}\mathrm{V}$ values. A total of 38.61% of the counties in agricultural zone I correspond to the highest $\mathrm{C}\mathrm{V}$ level, while 31.29% of the counties in agricultural zone VI correspond to the lowest $\mathrm{C}\mathrm{V}$ level (Table 1). A total of 29.41%, 24.4%, 32.14% and 40.98% of the counties in agricultural zones II, III, V and VIII had moderate $\mathrm{C}\mathrm{V}$ (see Table 1).

**Table 1:** Percentage of counties at each $\mathrm{C}\mathrm{V}$ level to the total counties in the corresponding main agricultural zones.

Level	CV	I	II	III	IV	V	VI	VII	VIII
Lowest	0.00–0.17	12.87	10.29	21.20	22.81	14.29	31.29	25.93	0.00
Lower	0.17–0.22	12.87	17.65	21.60	35.09	14.29	13.50	29.63	8.20
Moderate	0.22–0.28	16.83	29.41	24.40	14.04	32.14	24.54	18.52	40.98
Higher	0.28–0.33	18.81	14.71	19.60	12.28	25.00	15.34	14.81	22.95
Highest	0.33–0.63	38.61	27.94	13.20	15.79	14.29	15.34	11.11	27.87

### Distribution of risk index (${\mathbf{I}}_{\mathbf{R}}$)

**Figure 3:**
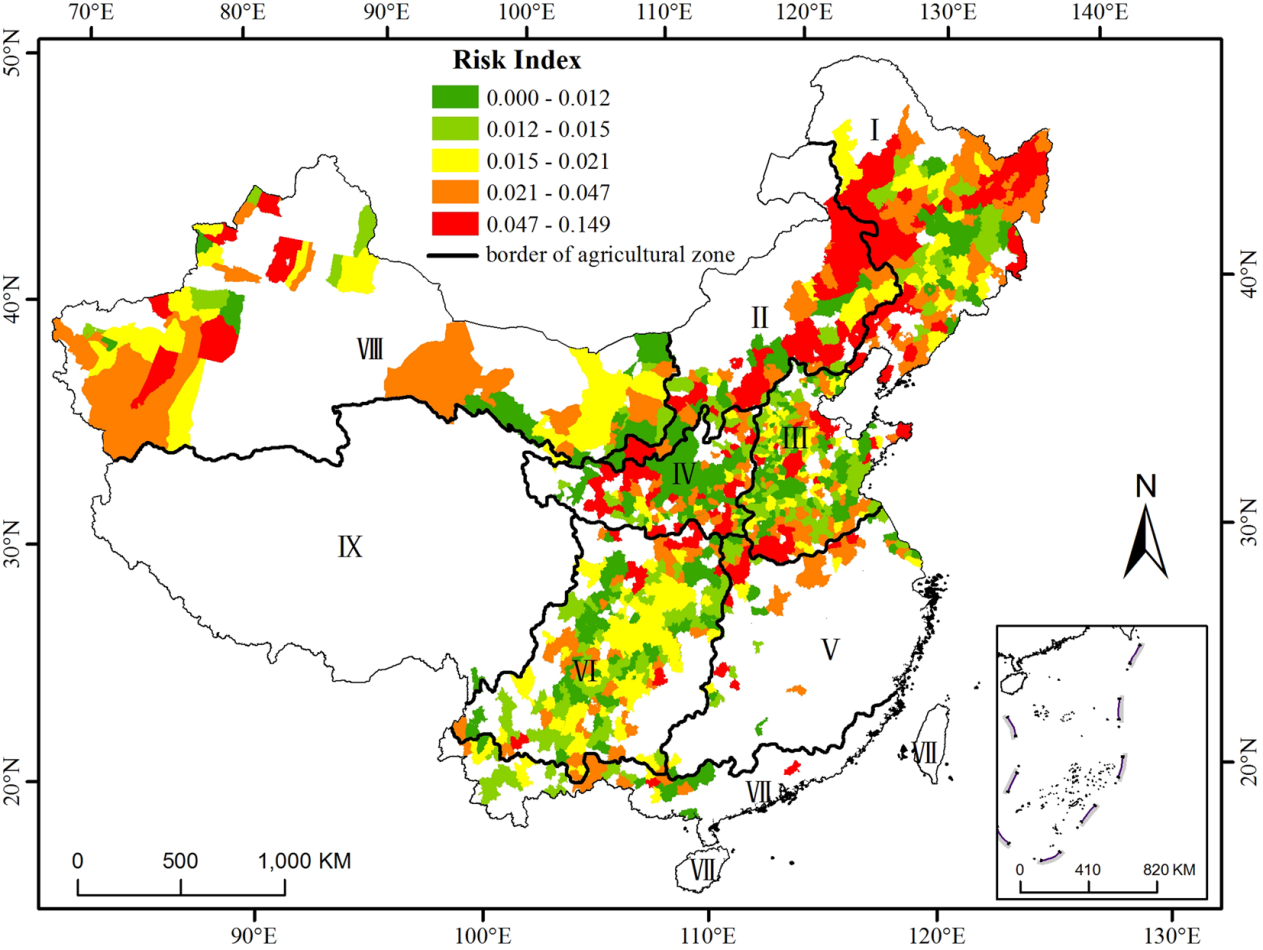
Distribution of the risk index (${\mathrm{I}}_{\mathrm{R}}$) of maize for the main production regions in China from 1981 to 2010. The data were processed in MATLAB software^32^ version R2016a (https://cn.mathworks.com/products/matlab/). The map was generated with ESRI ArcGIS software^33^ version 10.2.1 (https://www.esri.com/software/arcgis/arcgis-for-desktop).

The risk of maize yield loss increases with ${\mathrm{I}}_{\mathrm{R}}$, which means that the yield is vulnerable to climatic conditions. The two regions that were identified to exhibit a high risk covered (1) parts of Heilongjiang and Jilin Provinces and the eastern part of Inner Mongolia and (2) the western part of Xinjiang (Fig. 3). Agricultural zones I, II and IV had 38.61%, 57.35% and 24.56% of their counties in the highest ${\mathrm{I}}_{\mathrm{R}}$ level (see Table 2). Agricultural zones V and VII had 32.26% and 30% of their counties in the high ${\mathrm{I}}_{\mathrm{R}}$ level. Agricultural zone VI had more counties with the lowest risk, low risk, and moderate risk than the other zones (see Table 2).

**Table 2:** Percentage of counties at each level of ${\mathrm{I}}_{\mathrm{R}}$ to the total counties in the corresponding main agricultural zones.

Level	${\mathrm{I}}_{\mathrm{R}}$	I	II	III	IV	V	VI	VII	VIII
Lowest	0–0.012	8.91	7.35	21.60	22.81	19.35	19.63	16.67	18.46
Low	0.012–0.015	9.90	10.29	29.60	17.54	16.13	31.90	26.67	15.38
Moderate	0.015–0.021	16	7.35	20.80	11.40	12.90	25.15	20.00	35.38
High	0.021–0.047	25.74	17.65	16.00	23.68	32.26	17.18	30.00	20.00
Highest	0.047–0.149	38.61	57.35	12.00	24.56	19.35	6.13	6.67	10.77

The comparison of Figs. 2 with 3 indicates that there are some differences between the spatial distributions of the $\mathrm{C}\mathrm{V}$ and ${\mathrm{I}}_{\mathrm{R}}$. The reason for these differences might be that ${\mathrm{I}}_{\mathrm{R}}$ is related to only climatic conditions such as precipitation, temperature, sunshine hours and soil type. However, the $\mathrm{C}\mathrm{V}$ is related to the local socioeconomic conditions (technological developments, infrastructure and investment level) in addition to the climatic conditions.

### Performance of two comprehensive risk indices ($\mathbf{C}\mathbf{R}\mathbf{I}$ and $\mathbf{I}\mathbf{C}\mathbf{R}\mathbf{I}$)

**Figure 4:**
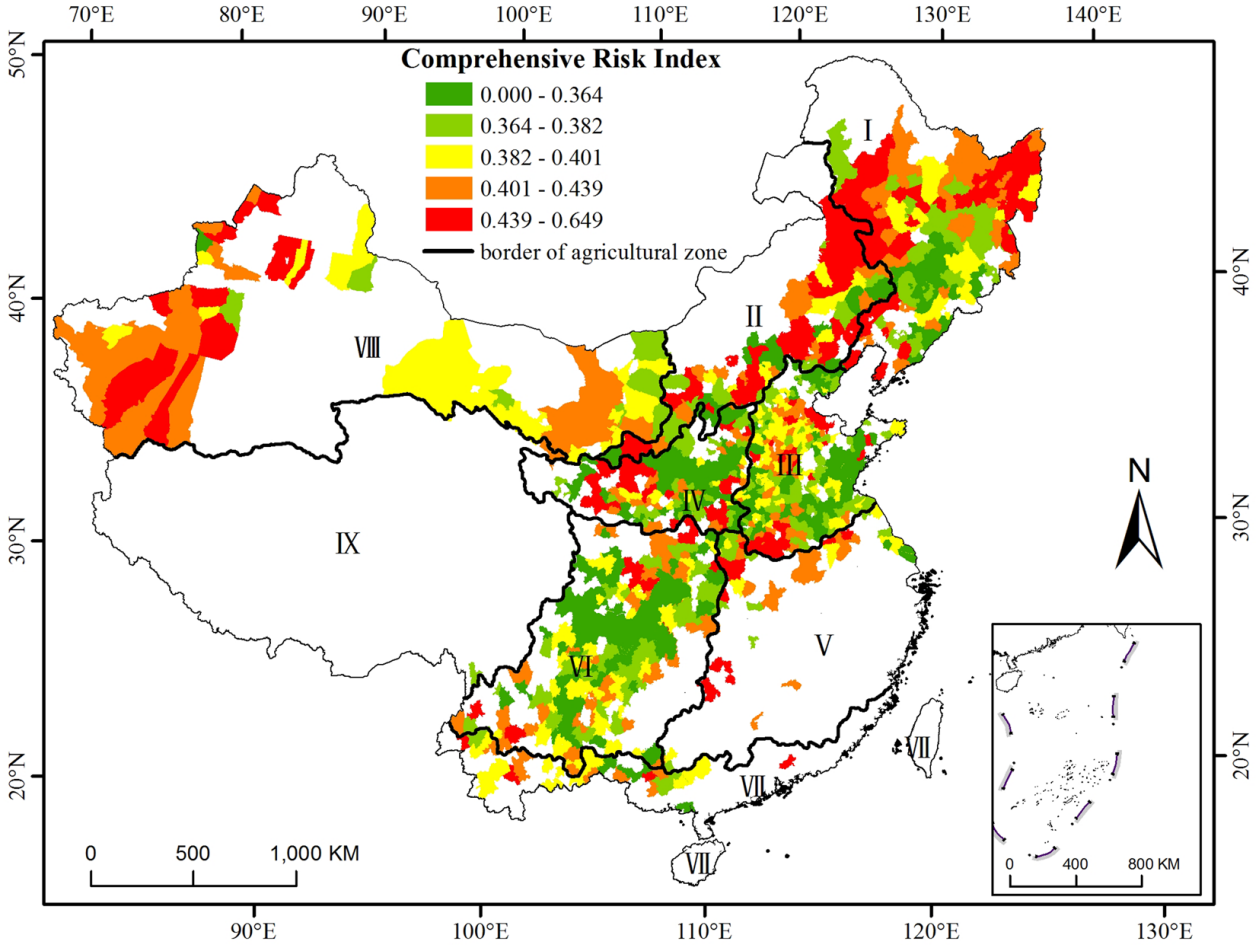
Distribution of the comprehensive risk index (CRI) of maize in the main production regions from 1981 to 2010 without removing the area effect. The data were processed in MATLAB software^32^ version R2016a (https://cn.mathworks.com/products/matlab/). The map was generated with ESRI ArcGIS software^33^ version 10.2.1 (https://www.esri.com/software/arcgis/arcgis-for-desktop).

**Figure 5:**
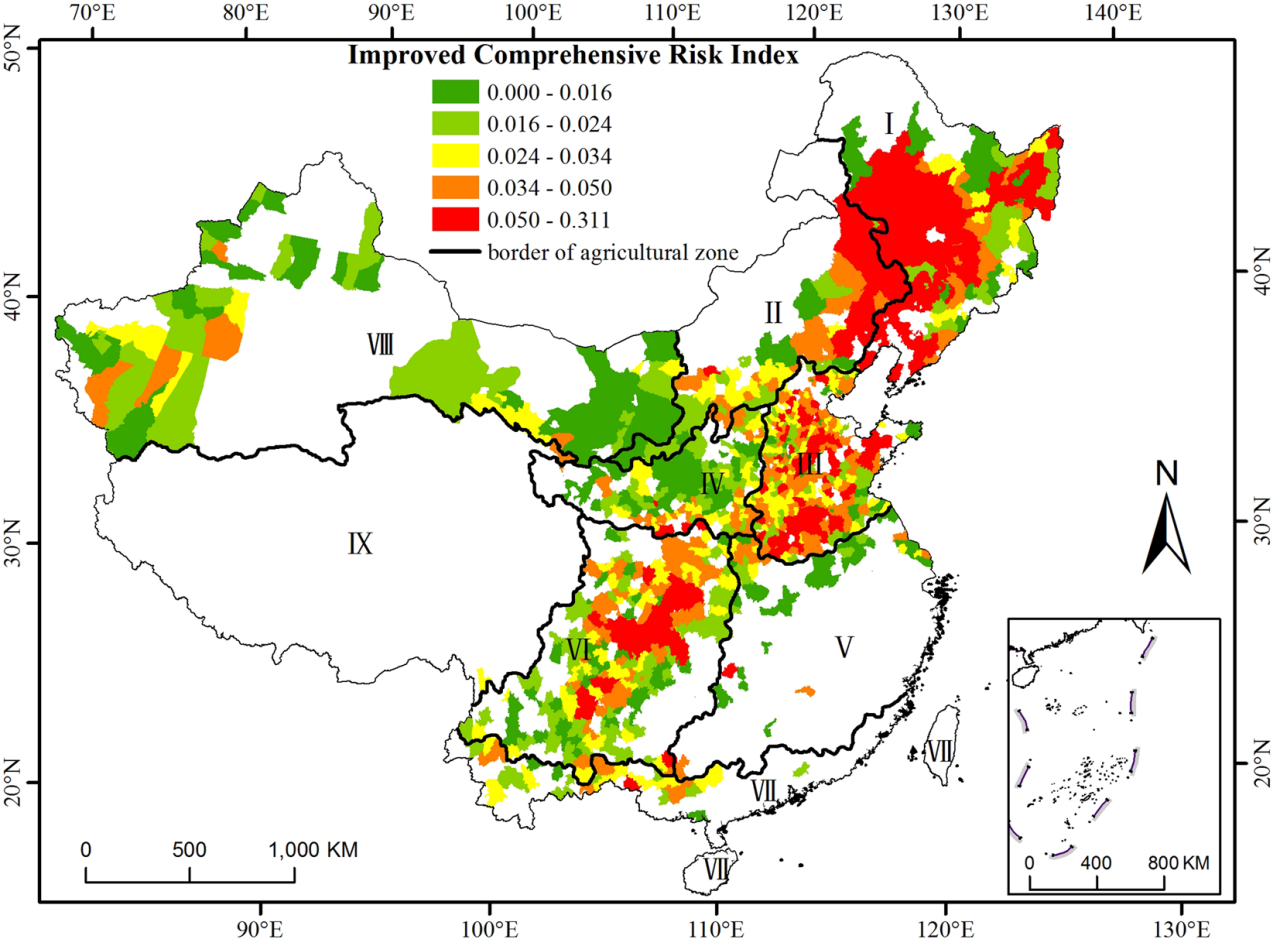
Distribution of the improved comprehensive risk index ($\mathrm{I}\mathrm{C}\mathrm{R}\mathrm{I}$) of maize in the main production regions from 1981 to 2010 after removing the area effect. The data were processed in MATLAB software^32^ version R2016a (https://cn.mathworks.com/products/matlab/). The map was generated with ESRI ArcGIS software^33^ version 10.2.1 (https://www.esri.com/software/arcgis/arcgis-for-desktop).

The risk of maize yield loss increases with the comprehensive risk index. A high value of the comprehensive risk index means that the maize yield is dramatically impacted by both climatic and socioeconomic conditions. Figures 4 and 5 show the distributions of the comprehensive risk index without considering the planting area effect and after removing the area effect, respectively. After the effect was removed, the improved comprehensive risk index $(\mathrm{I}\mathrm{C}\mathrm{R}\mathrm{I})$ identified three regions with high risk covering (1) parts of Heilongjiang and Jilin Provinces and the eastern part of Inner Mongolia, (2) parts of Hebei, Shandong, Henan and Anhui Provinces, and (3) the western central part of zone VI (Fig. 5). More counties in zones I, III, and III were at high risk after the area effect was removed, while the risk in zone VIII decreased dramatically, especially in the western (Xinjiang) and eastern (Hetao area) parts of zone VIII (Figs. 4 and 5). Across China, the degree of yield loss risk increased gradually from south to north and from east to west (Fig. 5). As shown in Table 3, 62.38%, 25.37% and 25.6% of the counties in agricultural zones I, II and III had the highest $\mathrm{I}\mathrm{C}\mathrm{R}\mathrm{I}$, respectively. A total of 37.72%, 58.62% and 50.82% of the counties in agricultural zones IV, V and VII had the lowest $\mathrm{I}\mathrm{C}\mathrm{R}\mathrm{I}$. A total of 27.6% and 34.62% of the counties in agricultural zones III and VII, respectively, had a moderate $\mathrm{I}\mathrm{C}\mathrm{R}\mathrm{I}$.

**Table 3:** Percentage of counties at each $\mathrm{I}\mathrm{C}\mathrm{R}\mathrm{I}$ level to the total counties in the corresponding main agricultural zones.

Level	$\mathrm{I}\mathrm{C}\mathrm{R}\mathrm{I}$	I	II	III	IV	V	VI	VII	VIII
Lowest	0–0.016	6.93	10.45	6.40	37.72	58.62	26.38	3.85	50.82
Low	0.016–0.024	6.93	22.39	14.40	28.07	13.79	28.83	38.46	32.79
Moderate	0.024–0.034	10.89	22.39	27.60	18.42	13.79	22.09	34.62	8.20
High	0.034–0.05	12.87	19.40	26.00	12.28	10.34	18.40	19.23	8.20
Highest	0.05–0.311	62.38	25.37	25.60	3.51	3.45	4.29	3.85	0.00

## Discussion

There are many different methods of crop risk assessment^17,24–27^. Quantifying the risk by the index calculated from historical time series yield data is often used^25–27^. Many indices, including the CV, reduction rate and probability of yield reduction, and a comprehensive index combining several indices, can indicate the fluctuation in crop yield^25,27,28^. However, the planting area of a county affects the risk assessment when field-based observed yields are aggregated at the county scale. As a consequence, the risk is underestimated or overestimated. In this study, the indicator of standardized planting area was introduced into the comprehensive risk index to remove this effect and improve the spatial comparability of the results.

In this research, natural and socioeconomic conditions were considered in the assessment of maize production risks. ${\mathrm{I}}_{\mathrm{R}}$ is related to only climatic conditions such as precipitation, temperature, sunshine hours and soil type, and the $\mathrm{C}\mathrm{V}$ indicates that the fluctuations between years are related to both local socioeconomic conditions (technological developments, infrastructure and investment level) and climate conditions, while the $\mathrm{I}\mathrm{C}\mathrm{R}\mathrm{I}$ integrates the $\mathrm{C}\mathrm{V}$, the average reduction rate and ${\mathrm{I}}_{\mathrm{R}}$ after removing the area effect. The $\mathrm{I}\mathrm{C}\mathrm{R}\mathrm{I}$ indicates the risk due to both climatic and socioeconomic conditions.

**Figure 6:**
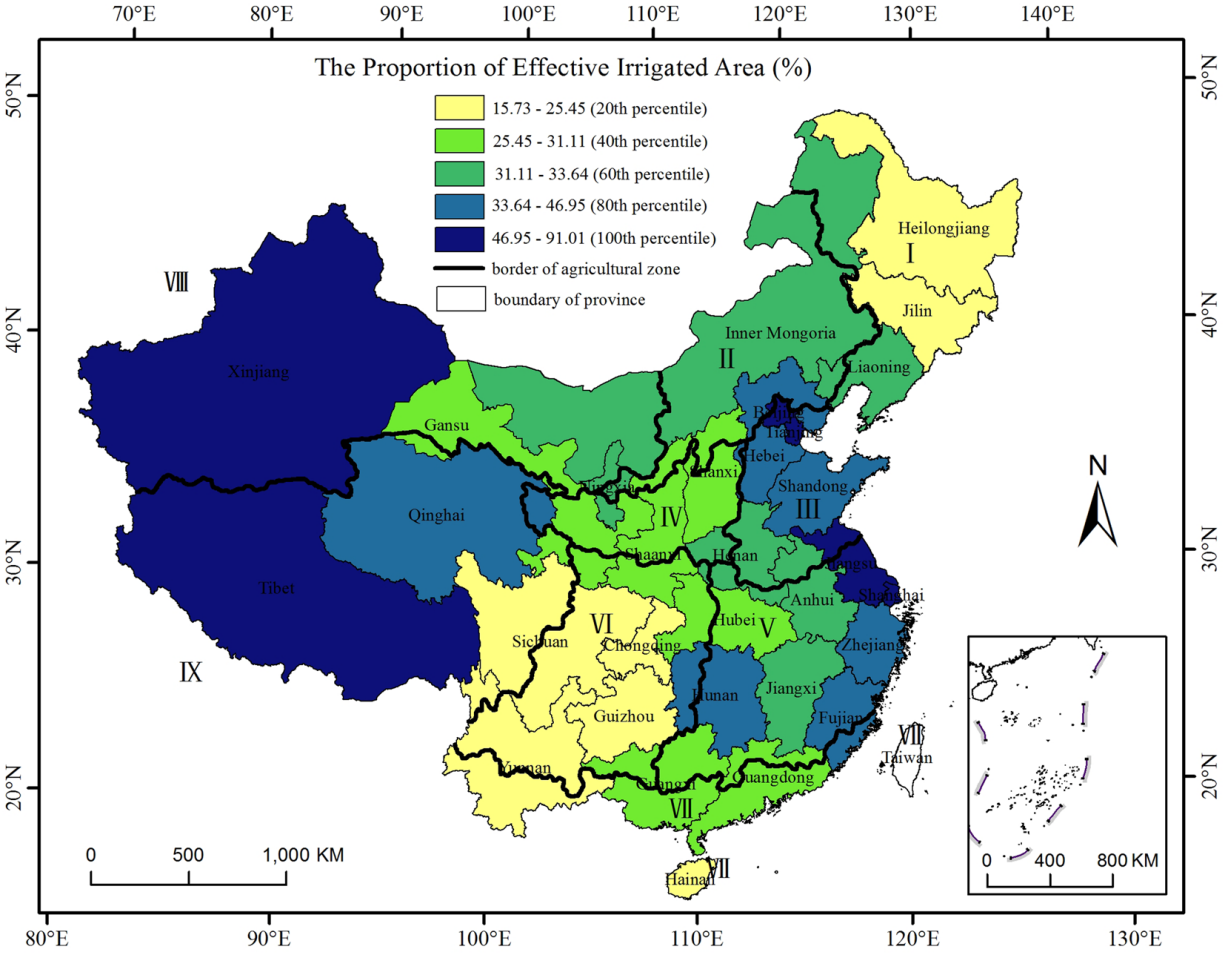
Distribution of the proportion of effective irrigated area to sown area at the provincial level. The data were processed in MATLAB software^32^ version R2016a (https://cn.mathworks.com/products/matlab/). The map was generated with ESRI ArcGIS software^33^ version 10.2.1 (URL: https://www.esri.com/software/arcgis/arcgis-for-desktop).

In agricultural zones I and II, including some parts of Heilongjiang, Jilin and Liaoning Provinces, and the eastern part of Inner Mongolia (the Inner Mongolia Plateau), there is a region with high $\mathrm{C}\mathrm{V}$, ${\mathrm{I}}_{\mathrm{R}}$ and $\mathrm{I}\mathrm{C}\mathrm{R}\mathrm{I}$ values, indicating that the maize yield in these areas varies considerably between years and that those areas are prone to disasters and changes in the socioeconomic level. Yield fluctuations increase from the south to the north in zone I^34,35^. This result occurs because the regions in these areas are rain-fed dryland cropping areas that are entirely dependent on limited and erratic rainfall^30^. The percentage of annual effective irrigated area to the sown area in Jilin and Heilongjiang Province accounted for no more than 26%, which was less than the 50th percentile of the proportion of all provinces (Fig. 6). A significant increase in the maize water requirements will occur in the future because precipitation has been continually decreasing, especially during the maize growing season^7,36,37^, while only approximately 6% of this region is irrigated^38^. Thus, drought is the greatest agro-meteorological disaster that occurs with the highest frequency, covers the largest area, and causes the most considerable agricultural production losses in this area^30^. The degree of drought disaster risk increases gradually from south to north and from east to west^29^, similar to the distribution of the yield loss risk. The areas that already correspond to a high yield loss risk will be significantly damaged by climate change. Therefore, the need for adjustment and management is urgent.

The $\mathrm{I}\mathrm{C}\mathrm{R}\mathrm{I}$ increased in most counties in zone III after the area effect was removed. The distribution of the $\mathrm{I}\mathrm{C}\mathrm{R}\mathrm{I}$ is consistent with the distribution of the drought disaster risk, which indicates a high risk in most areas in zone III, and the areas with the high risk and the highest risk accounted for more than 60% of the study area^12,30^. The high-risk areas of summer maize yield loss in Henan Province are distributed in the south and northwest parts of the province^39,40^, which is consistent with our results (Fig. 5, orange to red areas in the south part of zone III, north part of zone V, and southeast part of zone IV).

The yield loss risk increased significantly for some counties in zone VI, as indicated by the $\mathrm{I}\mathrm{C}\mathrm{R}\mathrm{I}$, because most areas of zone VI are rain-fed regions with a low proportion of effective irrigated area (Fig. 6). This zone is prone to spring and summer drought and heat stress, especially in the central and southwestern Sichuan Basin^6,41,42^, and the agricultural infrastructure is weak^30^.

The yield loss risk decreased in most counties in agricultural zone VIII. The sunshine and significant day/night temperature differences in this zone were suitable for maize growth^43^, although the annual precipitation was less than 400 mm/year (only 296 mm/year from 1981 to 2000) (Table 4). Thus, maize could not be grown in this area without irrigation^44,45^. In fact, irrigation is extensively used to relieve water shortages in these areas. The percentages of effective irrigated area to sown area accounted for 91.01% and 33.64% in Xinjiang and Inner Mongolia, respectively; these results are greater than the 50th percentile of the proportion of all provinces (Fig. 6). Thus, maize production in the western part (Xinjiang Province) and eastern part of zone VIII (Hetao area) had a low yield loss risk^6,46^. In turn, the ${\mathrm{I}}_{\mathrm{R}}$ was higher in these areas than in other areas, which was consistent with the trend of the average degree of drought hazard^34^, while the actual yield loss risk was lower, which indicated that the dependence on management such as irrigation cannot be underestimated.

**Table 4:** The annual average precipitation (P, mm), annual average temperature (T, ℃) in the 1981–2000 period, number of maize-growing counties (N), number of main maize-growing counties (N_m_), and percentage (%) of N_m_ to N in nine main agricultural zones.

ID	Name	P	T	N	N_m_	Percentage
I	The Northeast China	593	5.70	118	101	85.59
II	Inner Mongolia and Along the Great Wall	465	9.60	101	68	67.33
III	Yellow-Huai-Hai Zone	713	13.84	258	250	96.90
IV	Loess Plateau	449	10.77	172	114	66.28
V	Middle and Lower Reaches of Yangtze River	1,326	17.12	343	31	9.04
VI	The Southwest China	1,044	15.90	305	163	53.44
VII	The South China	1543	22.40	133	30	22.56
VIII	Gansu-Xinjiang	296	7.95	97	65	67.01
IX	Qinghai-Tibet	397	6.70	53	1	1.89

P and T in each zone were calculated by the spatial and temporal averages of the daily temperature and precipitation data.

Wind has played a significant role in maize yield loss^39,47^. Wind-induced lodging reduces the grain-filling rate and decreases the kernel weight^47,48^. In addition, the light penetration through the upper layer of vegetation is reduced by lodging^49^. It can decrease the ear number and kernel number per ear^48,50^. Lodging also can increase the yield loss of mechanical harvest^50,51^. Wind effects were not analyzed in our study but should be studied in the future.

## Conclusion

1. The area effect is the limiting factor for the spatial comparability of risk assessment because the planting area of a county affects the risk assessment when field-based or farm-based observed yields are aggregated by county.
2. High yield loss risk indicated by the ICRI occurs in (1) Northeast China, covering parts of Heilongjiang and Jilin Provinces and the eastern part of Inner Mongolia, (2) the Yellow-Huai-Hai Zone covering parts of Hebei, Shandong, Henan and Anhui Provinces, and (3) Southwest China, including the western central part. The Loess Plateau, middle and lower reaches of the Yangtze River and Gansu-Xinjiang are at low risk.
3. The distribution of yield loss risk after removing the area effect is consistent with the distribution of disaster risk (e.g., drought) and socioeconomic components (e.g., irrigation level); thus, the ICRI is reasonable. This index can be used to accurately compare the risk of maize yield loss in different areas.

## Data and methods

### Agricultural data and meteorological data

This study used the total yield (kg) and planting area (ha) of maize for 2,247 counties in China from 1981 to 2010; these data were collected by the Ministry of Agriculture and Rural Affairs of the People's Republic of China. The dataset contains summer maize and spring maize, while not all of the counties planted both types of maize. The yield per unit area was determined by the planting area and total yield. The counties used in this study were required to have at least fifteen records over the 1981–2010 period. The planting area sizes ranged from 2 ha in Lang County, Tibet, to 199,043 ha in Nongan County, Jilin Province.

The data of annual effective irrigated area and annual total sown area at the provincial level from 1981 to 2010 were collected by the National Bureau of Statistics (https://data.stats.gov.cn/); however, data were only available from 1996 to 2010 for Chongqing City.

The daily temperature and precipitation data from 740 stations during 1981–2000, which were collected by the China Meteorological Administration, were used.

### Study area

**Figure 7:**
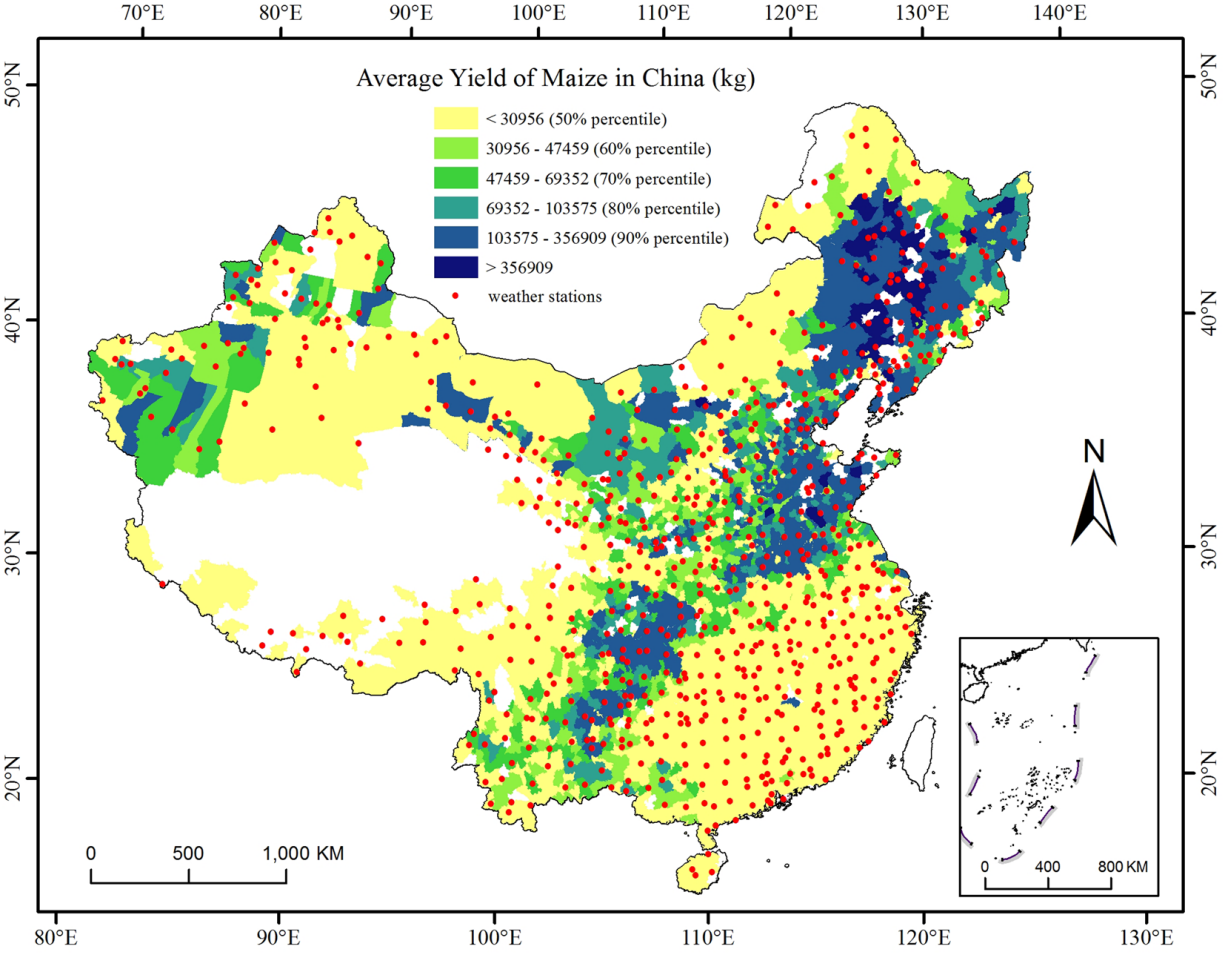
Location of 740 weather stations and distribution of the annual average total yield of maize at the county level in mainland China. The main production counties had an annual average total yield greater than the 50th percentile of the annual average total yield of all counties (light green to dark blue areas). The data were processed in MATLAB software^32^ version R2016a (https://cn.mathworks.com/products/matlab/). The map was generated in ESRI ArcGIS software^33^ version 10.2.1 (https://www.esri.com/software/arcgis/arcgis-for-desktop).

The study area included the main maize production regions in mainland China. The annual total yield in each county was calculated by the temporal average. The main production counties were those with an annual average total yield greater than the 50th percentile of the annual average total yield of all the counties. Finally, 823 counties were selected for analysis (in light green to dark blue areas, Fig. 7). There are nine main agricultural zones in mainland China^52^. The annual average precipitation (P, mm) and temperature (T, ℃) in each zone were calculated by the spatial and temporal averages of the daily temperature and precipitation data. The number of maize-growing counties (N), number of main maize-growing counties (N_m_), and percentage (%) of N_m_ to N in each zone are listed below (Table 4).

In this study, the observed maize yield per unit area (kg/ha) was used to obtain the CV. The yield per unit area time series was decomposed into three components by using the linear moving average method: the meteorological yield, the trending yield (or technical yield in some studies), and the error^53,54^. Ultimately, the meteorological yield was further processed to obtain the relative meteorological yield, in which the negative values were taken as the object to obtain the probability of reduction, risk index, and ICRI.

### Coefficient of variation ($\mathbf{C}\mathbf{V}$)

The CV of yield per unit area indicates the variations in yield caused by climatic and socioeconomic conditions. The equation is as follows:

1 $$\mathrm{C}\mathrm{V}=\sqrt{\frac{1}{\mathrm{n}-1}\times \sum_{\mathrm{i}=1}^{\mathrm{n}}({\mathrm{Y}}_{\mathrm{i}}-\overline{\mathrm{Y}})}$$

where ${\mathrm{Y}}_{\mathrm{i}}$ is the $\mathrm{i}$th observed yield per unit area (kg ha^−1^); $\overline{\mathrm{Y}}$ is the mean of ${\mathrm{Y}}_{\mathrm{i}}$ during the period 1981–2010; and $\mathrm{n}$ is the total number of observations, which is at least 15.

### Meteorological yield (${\mathbf{Y}}_{\mathbf{w}}$) and trending yield (${\mathbf{Y}}_{\mathbf{t}}$)

The observed maize yield per unit area is impacted by natural conditions (temperature and precipitation) and socioeconomic components (technological progress and infrastructure improvements). The yield can be divided into three parts: trending yield (${\mathrm{Y}}_{\mathrm{t}}$), meteorological yield (${\mathrm{Y}}_{\mathrm{w}}$) and random output/error ($\upvarepsilon$). The equation is as follows:

2 $$\mathrm{Y}={\mathrm{Y}}_{\mathrm{t}}+{\mathrm{Y}}_{\mathrm{w}}+\upvarepsilon$$

where $\mathrm{Y}$ is the annual observed maize yield per unit area. Since the random yield ε is quite small, it can be ignored. Furthermore, the simplified Eq. (1) is:

3 $$\mathrm{Y}={\mathrm{Y}}_{\mathrm{t}}+{\mathrm{Y}}_{\mathrm{w}}$$

The approach used to simulate the trending yield has an assumption that no marked technological progress took place in the time step chosen^55^. Although there is no definite evidence to show the time interval of the application of new crop varieties or technologies, the Five-Year Plan in China aims for economic growth and technological development. The period of research data (1981–2010) contains six Five-Year Plans, of which 1981 is the start of the 6th Five-Year Plan and 2010 is the end of the 11th Five-Year Plan. In addition, the trending yield and meteorological yield calculated with the 5-year linear moving average method met three criteria that determine trend models^56^, and this method can smooth irregularities and high-frequency variations in the trends^28^. Thus, the five-year linear moving average method was employed to simulate the trending yield. The time series of $\mathrm{Y}$ was divided into sequence segments according to the time step (k), which is 5 in this study. The number of segments is $\mathrm{n}-\mathrm{k}+1$. The linear regression for each segment ($\mathrm{j}$) is as follows:

4 $${\mathrm{Y}}_{\mathrm{j}}(\mathrm{t})={\mathrm{b}}_{\mathrm{j}}+{\mathrm{k}}_{\mathrm{j}}\times \mathrm{t}$$

5 $$\mathrm{t}=\left\{\begin{array}{c}1, 2, 3, \dots , k if j=1\\ 2, 3, 4, \dots , k+1 if j=2\\ \vdots \\ n-k+1, n-k+2, n-k+3, \dots , n if j=n-k+1\end{array}\right.$$

where ${\mathrm{Y}}_{\mathrm{j}}(\mathrm{t})$ is the ${\mathrm{tth}}$ trend yield in segment $\mathrm{j}$, ${\mathrm{k}}_{\mathrm{j}}$ and ${\mathrm{b}}_{\mathrm{j}}$ are estimated from a set of $\mathrm{Y}$ and $\mathrm{t}$ in segment $\mathrm{j}$ with the least squares method, and $\mathrm{t}$ is the rank index of each observed year. There can be more than one simulated value for each $\mathrm{t}$ in segment 2 to $\mathrm{n}-\mathrm{k}+1$. Finally, the trending yield of each $\mathrm{t}$ is a moving average:

6 $${\mathrm{Y}}_{\mathrm{t}}\left(\mathrm{t}\right)=average\left(\sum_{\mathrm{j}=1}^{\mathrm{n}-\mathrm{k}+1}{\mathrm{Y}}_{\mathrm{j}}\left(\mathrm{t}\right)\right) t=1, 2, 3, \dots , n$$

where ${\mathrm{Y}}_{\mathrm{t}}(\mathrm{t})$ is the ${t}{\text{th}}$ trending yield.

7 $${\mathrm{Y}}_{\mathrm{w}}(t)= \mathrm{Y}(\mathrm{t})-{\mathrm{Y}}_{\mathrm{t}}\left(\mathrm{t}\right)$$

where ${\mathrm{Y}}_{\mathrm{w}}(t)$ $\mathrm{Y}\left(\mathrm{t}\right)$ are the ${t}{\text{th}}$ meteorological yield and actual yield per unit area, respectively.

### Relative meteorological yield (${\mathbf{Y}}_{\mathbf{r}}$)

The ${\mathrm{Y}}_{\mathrm{r}}$ values are comparable since they are not impacted by the socioeconomic component^15^. The corresponding equation is as follows:

8 $${\mathrm{Y}}_{\mathrm{r}}(\mathrm{t})=\frac{{\mathrm{Y}}_{\mathrm{w}}(t)}{{\mathrm{Y}}_{\mathrm{t}}(t)}$$

where a negative ${\mathrm{Y}}_{\mathrm{r}}(\mathrm{t})$ is defined as the ${t}{\text{th}}$ reduction rate^3^.

### Average yield reduction rate ($\mathbf{R}$)

The average yield reduction rate ($\mathrm{R}$) was determined by the negative value of ${\mathrm{Y}}_{\mathrm{r}}(\mathrm{t})$. The corresponding equation is as follows:

9 $${\text{R}} = - \frac{1}{{\text{n}}} \times \mathop \sum \limits_{{{\text{i}} = 1}}^{{\text{n}}} {\text{Y}}_{{\text{r}}} \left( {\text{t}} \right)\;\;\;\;\;{\text{when}} \;{\text{Y}}_{{\text{r}}} \left( {\text{t}} \right) < \, 0$$

where $\mathrm{n}$ is the number of negative values of ${\mathrm{Y}}_{\mathrm{r}}(\mathrm{t})$.

### Risk index of yield loss (${\mathbf{I}}_{\mathbf{R}}$)

${\mathrm{I}}_{\mathrm{R}}$ results from the integration of different levels of reduction rates (${\mathrm{R}}_{\mathrm{i}}$) and their probability of occurrence (${\mathrm{P}}_{\mathrm{i}}$). The greater the value of ${\mathrm{I}}_{\mathrm{R}}$ is, the greater the risk of yield losses.

Because the climate factors that affect crop yield exhibit a normal distribution, it is argued that the ${\mathrm{Y}}_{\mathrm{r}}$ series should also exhibit a normal distribution. The normal distribution test was performed on ${\mathrm{Y}}_{\mathrm{r}}$ to verify this assumption. Due to the small sample size, the Lilliefors goodness-of-fit test was chosen. For a few samples that did not fit the normal distribution, the normal conversion was conducted by the logarithmic method.

There is no fixed standard for the division of the range of ${\mathrm{R}}_{\mathrm{i}}$. The China national standard (GB/T24438.1-2009) roughly divides ${\mathrm{R}}_{\mathrm{i}}$ into three ranges, 0.1–0.3, 0.3–0.8, and 0.8–1, to indicate three levels of damaged crop area. The threshold values of ${\mathrm{R}}_{\mathrm{i}}$ for identifying different levels of drought (mild, moderate, severe, and extreme drought) are 0.1, 0.2, and 0.3^57^. A value of 0.05 was used to determine whether the crop was impacted by a disaster^58^. Based on the above threshold, ${\mathrm{R}}_{\mathrm{i}}$ was divided into four ranges: $\left(0,\left. 0.05\right]\right.$, $\left(0.05,\left. 0.15\right]\right.$, $\left(0.15,\left.0.35\right]\right.$ and $\left(0.35,\left.1\right]\right.$. The equation for ${\mathrm{I}}_{\mathrm{R}}$ is as follows:

10 $${\mathrm{I}}_{\mathrm{R}}=\sum_{\mathrm{i}=1}^{\mathrm{n}}\left({\mathrm{R}}_{\mathrm{i}}\times {\mathrm{P}}_{\mathrm{i}}\right)$$

### Comprehensive risk index of yield loss ($\mathbf{C}\mathbf{R}\mathbf{I}$)

The $\mathrm{C}\mathrm{R}\mathrm{I}$ combines $\mathrm{C}\mathrm{V}$, $\mathrm{R}$, and ${\mathrm{I}}_{R}$. A larger $\mathrm{C}\mathrm{R}\mathrm{I}$ means a greater risk of losses. Due to the inconsistent units of the four variables, standardization is first performed using the extreme difference method. The standardized $\mathrm{C}\mathrm{V}$, $\mathrm{R}$, and ${\mathrm{I}}_{R}$ were calculated using the following equation:

11 $${\mathrm{x}}_{\mathrm{s}}=\frac{\mathrm{x}-\mathrm{m}\mathrm{i}\mathrm{n}(\mathrm{x})}{\mathrm{m}\mathrm{a}\mathrm{x}(\mathrm{x})-\mathrm{m}\mathrm{i}\mathrm{n}(\mathrm{x})}$$

where $\mathrm{x}$ is $\mathrm{C}\mathrm{V}$/$\mathrm{R}$/${\mathrm{I}}_{R}$, and ${\mathrm{x}}_{\mathrm{s}}$ is the standardized $\mathrm{x}$.

$\mathrm{C}\mathrm{R}\mathrm{I}$ is the comprehensive risk index without considering the area effect.

12 $$\mathrm{C}\mathrm{R}\mathrm{I}=\frac{1}{3}\times \left({\mathrm{R}}_{\mathrm{s}}+{\mathrm{C}\mathrm{V}}_{\mathrm{s}}+{\mathrm{I}}_{\mathrm{R}\mathrm{s}}\right)$$

where ${\mathrm{C}\mathrm{V}}_{\mathrm{s}}$, ${\mathrm{R}}_{\mathrm{s}}$ and ${\mathrm{I}}_{\mathrm{R}\mathrm{s}}$ are the standardized versions of $\mathrm{C}\mathrm{V}$, $\mathrm{R}$, and ${\mathrm{I}}_{R}$. The weights of these three indicators are the same^28,58,59^.

### Improved comprehensive risk index ($\mathbf{I}\mathbf{C}\mathbf{R}\mathbf{I}$) of yield loss

$\mathrm{R}$, $\mathrm{C}\mathrm{V}$ and ${\mathrm{I}}_{\mathrm{R}}$ exhibit close positive correlations with the yield loss risk, while planting area size ($\mathrm{S}$) exhibits a negative correlation with this risk because the increase/decrease in the yield of a field will offset the decrease/increase in another field in the same region. The $\mathrm{I}\mathrm{C}\mathrm{R}\mathrm{I}$ is the comprehensive risk index after removing the planting area effect. The main maize growing counties were divided into lowest-, low-, moderate-, high- and highest-risk areas.

The $\mathrm{I}\mathrm{C}\mathrm{R}\mathrm{I}$ equation is as follows:

13 $$\mathrm{I}\mathrm{C}\mathrm{R}\mathrm{I}=\frac{1}{3}\times \left({\mathrm{R}}_{\mathrm{s}}+{\mathrm{C}\mathrm{V}}_{\mathrm{s}}+{\mathrm{I}}_{\mathrm{s}}\right)\times \frac{1}{{S}_{s}}$$

where ${\mathrm{S}}_{\mathrm{S}}$ is the standardized planting area size calculated using Eq. (11).

## Data Availability

All the data analyzed in this study are included in this published article. The original data are available from the corresponding author upon reasonable request.
